# The impact of close surgical margins on recurrence in oral squamous cell carcinoma

**DOI:** 10.1186/s40463-020-00483-w

**Published:** 2021-02-12

**Authors:** Joseph Solomon, Ashley Hinther, T. Wayne Matthews, Steven C. Nakoneshny, Rob Hart, Joseph C. Dort, Shamir P. Chandarana

**Affiliations:** 1 Cumming School of Medicine, University of Calgary, Calgary, Canada; 2 Department of Surgery, Section of Otolaryngology – Head & Neck Surgery, Calgary, Canada; 3 Ohlson Research Initiative, Arnie Charbonneau Cancer Institute, Cumming School of Medicine, University of Calgary, Calgary, Canada; 4 Foothills Medical Centre, North Tower Rm 1012, 1403 29 St NW, Calgary, AB T2N 2T9 Canada

**Keywords:** Margin, Oral cavity, Squamous cell carcinoma, Recurrence, Pathology

## Abstract

**Background:**

Close margins influence treatment and outcome in patients with oral squamous cell carcinoma (OSCC). This study evaluates 187 cases of surgically treated OSCC regarding the impact of close margins on recurrence-free survival (RFS) and disease-specific survival (DSS).

**Methods:**

Predictors of worsened outcome were identified using Kaplan-Meier analysis and multivariate Cox regression analysis.

**Results:**

Tumour size [HR:1.70(0.95–3.08)], nodal status [HR:2.15(1.00–4.64)], presence of extracapsular spread (ECS) [HR:6.36(2.41–16.74)] and smoking history [HR:2.87(1.19–6.86)] were associated with worsened RFS. Similar factors were associated with worsened DSS. Close margins did not influence RFS or DSS.

**Conclusions:**

While most conventional risk factors for OSCC conferred a worsened outcome, close margins did not. One explanation for this would be that close margins (< 5 mm) are equivalent to clear margins and the cutoff definition for a close margin should be re-evaluated. Lack of standardized pathology could also reduce accuracy of reporting of close surgical margins.

**Graphical abstract:**

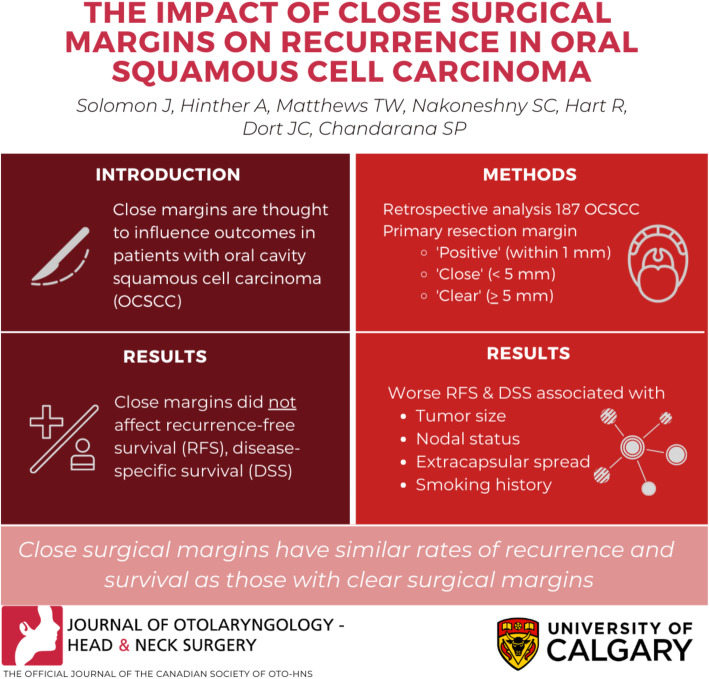

## Background

Surgical margin status in oral squamous cell carcinomas (OSCC) is felt to predict both recurrence rate and long-term patient survival [1–5]. A surgical margin of 5 mm or greater has been correlated with better local control and disease specific survival [3]. Margin status is not only used to determine patient prognosis but is also used to guide the use of adjuvant treatment such as radiation therapy, systemic chemotherapy or revision surgery. Due to the impact of achieving a clear margin, an accurate interpretation of the pathologic surgical margin is necessary to optimize patient management.

OSCC surgical margins are particularly difficult to interpret due to the complex three-dimensional anatomy of the oral cavity subsites, the handling of the specimen from resection to interpretation, and tissue shrinkage immediately post operatively and after fixation [6–8]. As a result, a dedicated team of head and neck specialists, including surgeons and pathologists, are essential to the functioning multidisciplinary team treating OSCCs.

At the University of Calgary, we have implemented a formal clinical outcomes assessment program that tracks and reports a number of clinical and process outcome measures. As part of our routine surgical quality assurance evaluation we noted that a high proportion of patients undergoing surgery for OSCC had close (< 5 mm) surgical margins. This observation was concerning and was therefore felt to warrant further study. The aim of this study was to determine which independent patient and tumour factors predicted worsened recurrence rate and disease-free survival, in a homogenous population of surgically treated patients with OSCC. Specifically, we sought to determine if margins reported as close (< 5 mm) were associated with a higher likelihood of recurrence and worsened disease-specific survival. Based on our known OSCC clinical outcomes we hypothesized that close surgical margins would not be associated with worsened outcome.

## Methods

**Fig. 1:**
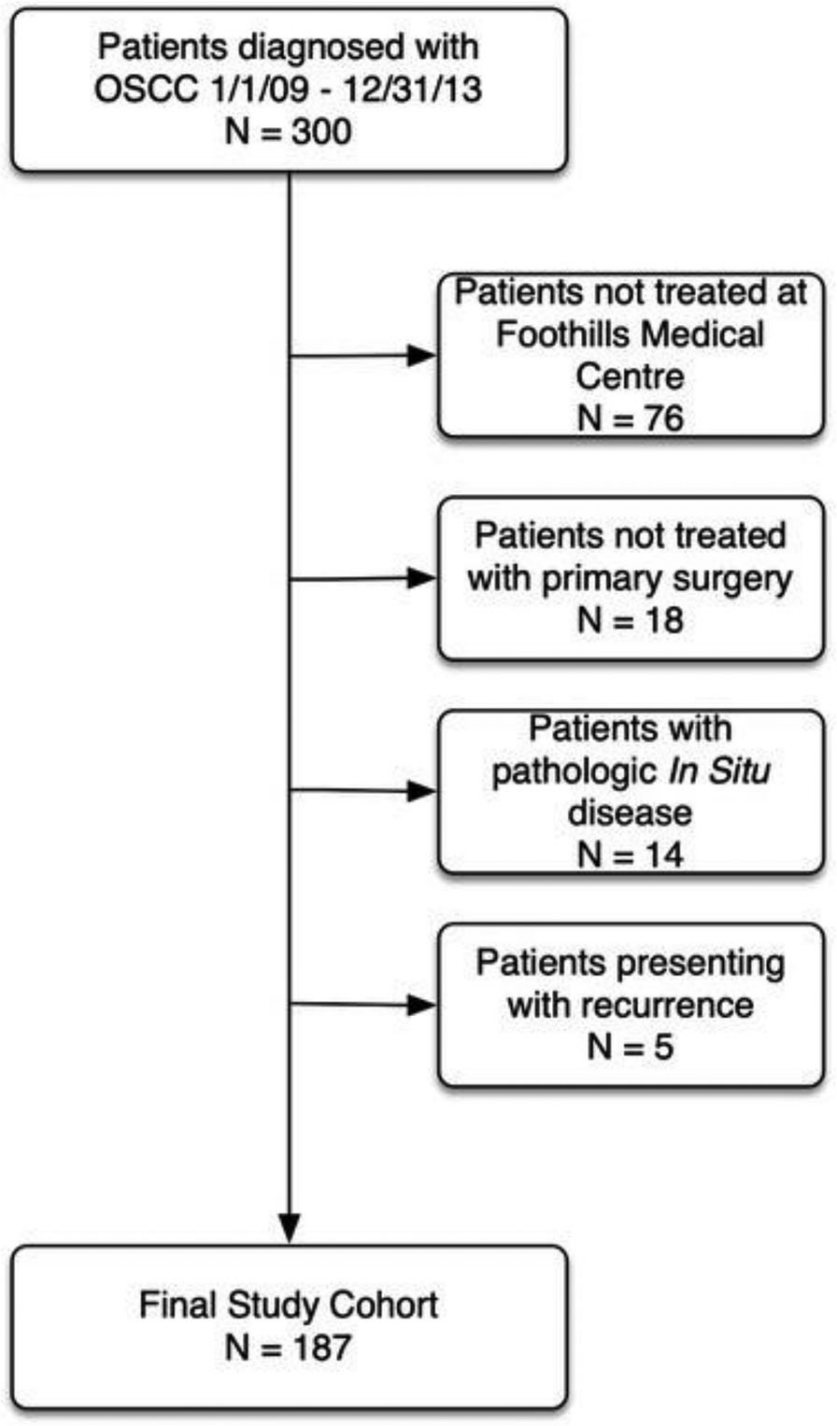
Patient Inclusion/Exclusion Criteria

All adult patients (age 18 or older) that underwent primary surgical resection of OSCC were eligible for this study. The prospective cohort included 300 patients treated between the dates of January 1, 2009 – December 31, 2013 at a major tertiary care hospital by three head and neck surgeons. Patients presenting with recurrent OSCC, a second primary malignancy, a synchronous primary malignancy, or who did not receive surgery as a primary treatment modality were excluded. Figure 1 outlines the number and reasons for patient exclusion. After exclusions, the final study cohort included 187 patients and mean follow-up time was 21.5 months. Of the 187 patients with OSCC, 90 arose from the tongue, 34 from the floor of mouth, 23 from the mandibular/maxillary alveolus/gingiva, and 40 from other subsites of the oral cavity (not specified).

Information was prospectively collected on patient demographic and risk factors (age, gender, and smoking status), pathologic factors (T – stage, N – stage, margin status taken from primary tumor specimen, presence of extracapsular spread within lymph nodes [ECS], lymphovascular invasion [LVI], perineural invasion [PNI]) and treatment factors (administration of adjuvant radiotherapy).

Tumours were classified according to the TNM staging system using the seventh edition of the American Joint Committee on Cancer staging manual [9]. At the time of surgery, frozen section margins were taken from the resultant tumour bed, after removal of the tumour specimen. Final surgical margin status was based on evaluation of the primary tumor specimen and defined as positive (evidence of malignancy within 1 mm from surgical margin), close (less than 5 mm but not positive at surgical margin) or clear (greater than or equal to 5 mm).

Categorical outcomes were compared using either chi-square or Fisher’s exact test as appropriate. Continuous outcomes were compared using either Student’s t-test or the Wilcoxon rank-sum test as appropriate. Recurrence-free survival (RFS) as well as disease-specific survival (DSS) were determined by comparing the time-to-event (Kaplan-Meier survival curves) of the pre-defined groups of interest using a log-rank test statistic. In addition, the evaluation of RFS and DSS between groups, with adjustment for confounding variables (tumour stage, smoking, etc.) was carried out using Cox proportional hazards (PH) regression models. Key assumptions for these models, such as the proportionality of the hazards and the functional form of the continuous variables were assessed. All final multivariable Cox PH regression models were evaluated for goodness-of fit, model stability and influential observations. A *p*-value < 0.05 was considered statistically significant. Statistical Analysis was performed using Stata, version 14 (Stata Corp. College Station, Tx, USA).

The study was approved by the University of Calgary Conjoint Health Research Ethics Board.

## Results

Table 1 demonstrates relevant patient and tumour characteristics, stratified by whether patients did or did not receive postoperative adjuvant treatment. Of the 187 patients, 112 received surgery alone, 56 received surgery + radiotherapy, and 19 received surgery + chemoradiotherapy. Patients who received adjuvant treatment were more likely to have adverse risk factors such as smoking (*p* = .01), advanced T-stage (*p* = .01), advanced N-stage (*p* = .01), presence of ECS (*p* = .01), LVI (*p* = .01) and PNI (*p* = .01). Furthermore, patients with close surgical margins were more likely to receive adjuvant treatment (*p* = .01).

**Table 1:** Clinical patient characteristics

	Surgery Alone ***n*** = 112 (60%)	Surgery + Adjuvant ***n*** = 75 (40%)	***P***-value
**GENDER**			NS
Male	64 (34%)	51 (27%)	
Female	48 (26%)	24 (13%)	
**Age (mean)**	61	62	NS
**Risk Factors**			.01
Never smoker	36 (20%)	12 (6%)	
Smoker	74 (40%)	65 (34%)	
**TNM STAGING**			
Low T (1 and 2)	94 (50%)	32 (17%)	.01
High T (3 and 4)	18 (10%)	43 (23%)	
N negative	94 (50%)	21 (11%)	.01
N positive	16 (9%)	33 (18%)	
N positive w/ ECS	2 (1%)	21 (11%)	
**Cancer Stage**			.01
Low (stage 1/2)	81 (43%)	3 (2%)	
High (stage 3/4)	31 (17%)	72 (38%)	
**LVI**			.01
Yes	3 (2%)	13 (6%)	
No	90 (48%)	53 (29%)	
Not reported	19 (10%)	9 (5%)	
**PNI**			.01
Yes	10 (5%)	33 (18%)	
No	79 (42%)	34 (18%)	
Not Reported	23 (13%)	8 (4%)	
**Final Margin**			.01
Negative	70 (37%)	35 (18%)	
Close	36 (19%)	34 (18%)	
Positive	1 (1%)	5 (3%)	
Not reported	5 (3%)	1 (1%)	

### Recurrence-free survival

**Fig. 2:**
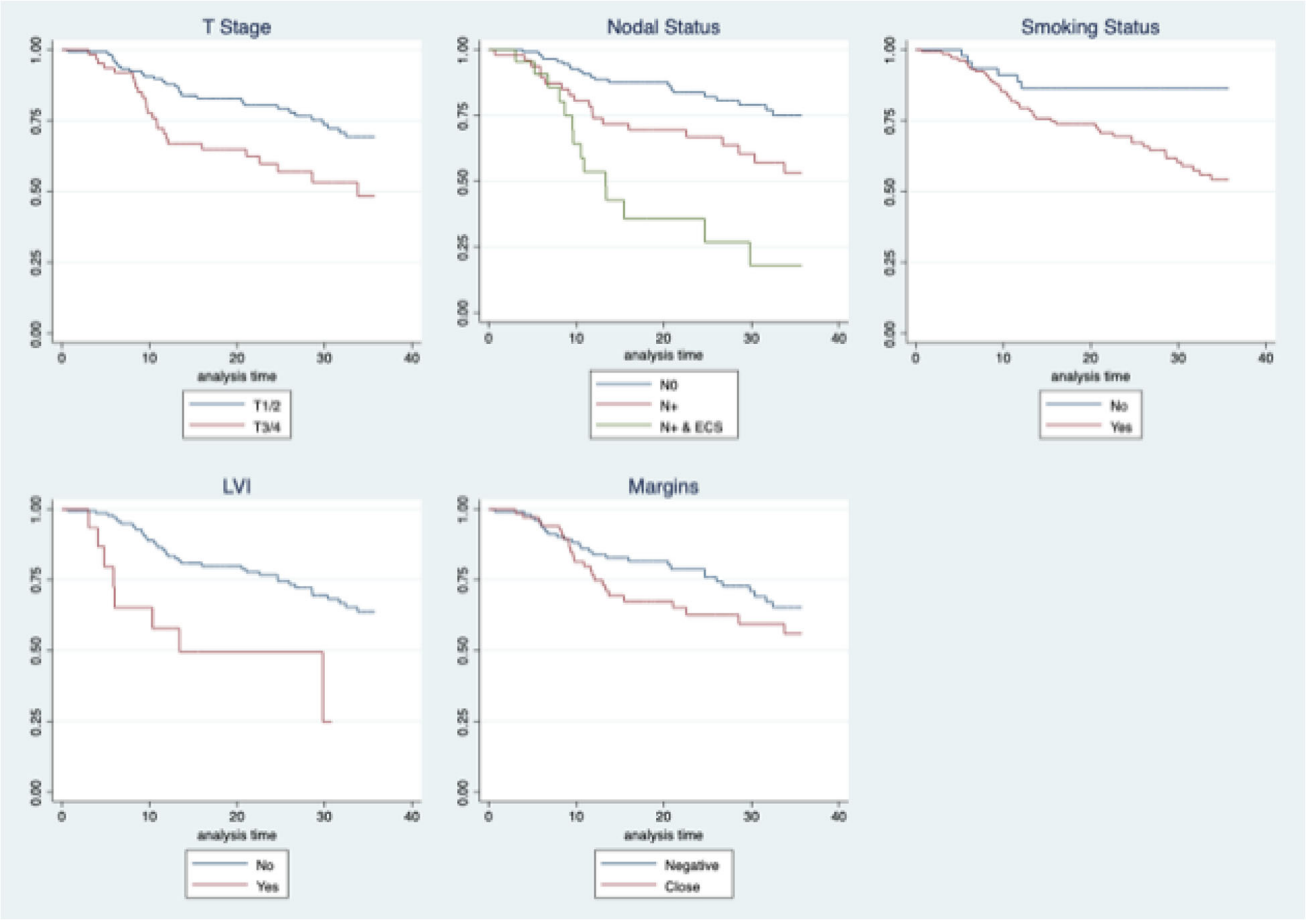
Recurrence Free Survival stratified by univariate factors. (N0 = No metastatic lymph nodes, N+ = positive lymph node metastases, ECS = lymph node with extracapsular extension, LVI = lymphovascular invasion of primary tumour

Univariate Kaplan-Meier analysis revealed that worse recurrence-free survival was associated with advanced T-stage [HR = 2.10 (1.22–3.60)], advanced N-stage [HR = 2.28 (1.22–4.24)], presence of ECS [HR = 5.80 (2.92–11.50)], smoking status [HR = 3.14 (1.34–7.35)], and presence of LVI [HR = 3.40 (1.57–7.35)]. The use of adjuvant treatment was also associated with worse RFS [HR = 2.43 (1.41–4.18)]. Margin status was not associated with RFS (Fig. 2).

After adjusting for the above-mentioned covariates on multivariate analysis, advanced T-stage [HR = 1.70 (.95–3.08)], advanced N-stage [HR = 2.15 (1.00–4.64)], presence of ECS [HR = 6.36 (2.41–16.74)], and smoking status [HR = 2.87 (1.19–6.86)] independently predicted worse RFS (Table 2). After multivariable adjustment, surgical margin status was not associated with worsened RFS. The use of adjuvant treatment (or lack thereof) was also not associated with RFS.

**Table 2:** Multivariate analysis (Cox regression) for Recurrence Free Survival

	Hazard ratio	***P***-value
**T- Classification**	1.70 (0.95–3.08)	.076
**N-Classification**		
Node positive/ECS neg	2.15 (1.00–4.64)	.05
Node positive/ECS pos	6.36 (2.41–16.74)	.000
**Smoking Status**	2.87 (1.19–6.86)	.018
**Margin Status**		
Close Margin	.97 (0.54–1.74)	NS
Positive Margin	1.22 (0.27–5.60)	NS
**Treatment Modality**	1.21 (0.56–2.65)	NS
(Surgery vs. Surgery/RT)		

### Disease-specific survival

On univariate Kaplan-Meier analysis, worsened disease-specific survival was associated with advanced T-stage [OR = 2.05 (1.11–3.80)], advanced N-stage [HR = 2.9 (1.38–6.10), presence of ECS [HR = 8.31 (3.81–18.1)], smoking [HR = 4.96 (1.53–16.1)], presence of LVI [HR = 4.72 (2.11–10.5)], and presence of PNI [HR = 2.57 (1.29–5.12)]. The use of adjuvant treatment was also associated with worse DSS [HR = 3.98 (2.03–7.81)]. Margin status was not associated with DSS.

After adjusting for the above-mentioned factors on multivariate analysis, presence of advanced N-stage with ECS [HR = 5.75 (1.97–16.80) and smoking status [HR = 4.16 (1.25–13.9)] remained significantly associated with worsened DSS (Table 3). Of note, after controlling for the above factors, surgical margin status was not associated with worsened DSS. The use of adjuvant treatment (or lack thereof) was also not associated with DSS.

**Table 3:** Multivariate analysis (Cox regression) for Disease Specific Survival

	Hazard ratio	***P***-value
**T- Classification**	1.64 (0.85–3.19)	NS
**N-Classification**		
Node positive/ECS neg	1.91 (0.77–4.81)	NS
Node positive/ECS pos	5.75 (1.97–16.80)	.001
**Smoking Status**	4.15 (1.25–13.9)	.02
**Margin Status**		
Close Margin	.95 (0.50–1.83)	NS
Positive Margin	.75 (0.09–6.12)	NS
**Treatment Modality**	.74 (0.28–1.88)	NS
(Surgery vs. Surgery/RT)		

A total of 32 pathologists reported on surgical specimens in this cohort of 187 patients treated over a 5-year period. The majority of pathologists interpreted fewer than 10 surgical specimens.

## Discussion

Current management of OSCC involves the combined efforts of highly skilled surgeons, radiation oncologists, medical oncologists, pathologists and other members of a multidisciplinary team. Current clinical practice guidelines indicate that, when feasible, surgery is the primary treatment for patients with resectable OSCC. Surgical margin status, among other factors, determines the use of adjuvant treatment such as RT and/or chemotherapy, based on the assumption that close margin status correlates with a worse patient prognosis. Indeed, in this study, patients with close margins were more likely to receive adjuvant RT.

This study agrees with current evidence demonstrating that conventional risk factors for OSCC, such as tumour size, lymph node status, presence of extracapsular spread, and smoking status all correlate with worsened recurrence-free survival and disease-specific survival [1, 2, 10]. However, a major goal of this study was to evaluate the impact of close (< 5 mm) surgical margins in OSCC. We found there was no correlation between close surgical margins and recurrence-free survival or disease-specific survival which is consistent with previous reports in the literature. One possible confounder could be that patients with adverse risk factors received adjuvant treatment, thus skewing the results toward a better outcome in this otherwise less-favourable group. While close margins was not the major indication for adjuvant treatment, patients with close margins were more likely to receive adjuvant treatment in this cohort. To control for this confounder, the administration of adjuvant treatment was included in the multivariate model. There was no correlation observed between administration of adjuvant treatment and outcome, which would suggest that administration of adjuvant treatment did not confer a survival advantage/disadvantage to those that received it, helping to reduce the impact of adjuvant treatment as a confounder of outcome in those with close margins.

The impact of the status of the resection margin (positive or negative) on survival outcomes of patients treated surgically for oral cancer has been reported in the literature [11–14]. However, the definition, as well impact, of a close surgical margin is not clear. The notion that a close surgical margin portends a worsened prognosis is based on multiple sources [1, 3, 4]. Many studies have examined the best cutoff point for margin distance and report a linear relationship between increasing margin distance and improved outcome [4, 15, 16]. In contrast, other studies, similar to ours, have shown that close surgical margins have no effect on outcome and therefore should not be used as the only factor in making decisions on adjuvant therapy [17, 18]. Ultimately, a wide range of cut-points for close margins have been reported ranging from 1 to 7 mm in oral cavity cancer. Liao et al. studied local tumour control and determined a margin < 7 mm to be a poor prognostic factor for local control [19]. Tasche et al. reported a cutoff margin of only < 1 mm that identified patients at increased risk of local recurrence [20]. Zanoni et al. determined that a significantly higher rate of local recurrence was seen when margins were < 2.2 mm, while patients with margins 2.3–5.0 mm had similar local recurrence free survival as those patients with tumour margins > 5.0 mm [21]. In our study, a discreet measurement to the nearest margin was inconsistently reported, and as such, deriving a specific cut point that correlated with worsened outcome was not a goal of this study. Nonetheless our study aligns with previous reports in that it would appear that there is a distinct difference in outcome when comparing a positive and a close margin, but that difference is less evident when comparing a close margin to a clear margin.

Other studies in the literature have examined the significance of resection margin but the data is confounded by factors such as unknown neck status and not adjusting for patients who have received post-operative radiotherapy; however, these studies do all support the need to reevaluate the definition of a close margin [4, 22]. In addition, durable long-term outcomes in the surgical management of oropharyngeal SCC via transoral approaches, where close but clear margins are frequent, also emphasize the need to reevaluate the impact of close margins. There is consensus on the fact that a truly positive margin (tumor has been cut through, leaving tumor behind in the resection bed) correlates with worsened recurrence rates and survival outcomes [1]. In our study we were unable to validate the effect of a true positive margin because the incidence of true positive margin in this cohort was so low.

Variability in surgical specimen handling and interpretation may be another reason why close surgical margins did not correlate with worsened outcome, Johnson et al. highlight the difficulties in measuring margins accurately during the initial stages of assessing the surgical specimen, noting that there is significant variability in the evaluation of surgical margins by pathologists due to handling of the specimen, formalin fixation and tissue shrinkage [7, 23]. Formalin induced shrinkage can be as much as 15% but varies by location of the specimen. An analysis by Batsakis et al., showed that a tongue specimen with a predetermined margin of 12 mm had reduced to 8.3 mm once the specimen was prepared; additionally, a buccal margin of 12 mm had diminished to 6.3 mm once fully prepared for viewing under a slide [7]. Johnson et al. found that the mean shrinkage of lingual surface mucosal margins was 30.7% (*p* < 0.0001), deep tongue margins shrank 34.5% (*p* < 0.0001) and labio-buccal mucosal margin shrinkage was 47.3% (*p* < 0.0001). This group concluded that an in vivo margin of 8 to 10 mm, not 5 mm would need to be taken to achieve the margin of 5 mm under a slide [23]. This reflects the current standard of practice at our institution, whereby margins of at least 10 mm are outlined around the tumour prior to resection. Most literature supporting the impact of a 5 mm margin made that determination after shrinkage had occurred. However, it is important to note that the amount of shrinking may vary from center to center depending on preparation, technique and site of resection.

In addition to the variability at the macroscopic level, there is lack of consensus on the definition of the microscopic margin of the tumour specimen. Depending on the author(s), the true margin may include any or all of the following: the border at which there is no *invasive* cancer, the border at which there is no *carcinoma-*in-situ, or the border where there is no *dysplasia* [7, 24]. A survey of the international American Head and Neck Society members with 476 respondents showed that there was no uniform criterion defining a margin status and that any mix of the 3 criteria listed above may be used in determining margin status [25].

In our centre, during the time period of this study, there were no pathologists dedicated to the interpretation of head and neck surgical specimens. Instead, head and neck pathologic specimens were evaluated by any one of a large number of pathologists, including the handling and preparation of the specimen, as well as the microscopic interpretation. Current studies on head and neck pathology show that inter-observer agreement between pathologists is poor to moderate, especially with more complex surgical specimens [7, 26–29] A discordance rate of 1–78% in the diagnosis of dysplasia by multiple pathologists has been shown by Pindborg et al. while Abbey et al. found a discordance rate of 42–62% with the grading of dysplasia [24, 27]. Multiple other studies confirm this finding of poor to moderate agreement in determining dysplasia with a great deal of subjectivity from one pathologist to another [6, 8, 28, 30, 31]. This was an issue in our centre, where there was no dedicated team of head neck pathologists involved in interpreting surgical margins over the 4-year study period. Pulling the specimens for all 187 patients, to allow for one index pathologist to independently review the slides for this study would not be feasible. Many of the specimens were ten or more years old, and acquiring these specimens was not an option. This, therefore, remains a limitation of the retrospective design of this study. Unfortunately, the lack of a dedicated head and neck pathologist was not unique to our centre. While many regional centres in Canada do have dedicated pathologist(s), there remain some centres that do not. Fortunately, more recently, and in large part to due to the findings of this study, our centre was able to successfully recruit and assemble a dedicated team of head and neck pathologists, and this team handles all head and neck surgical specimens.

There is now a call for a new, more concrete, guideline that better defines the criteria for a surgical margin, in order to reduce the heterogeneity of reporting [26, 32]. Such a guideline would better define what constitutes a true cancerous margin. It would also recommend having a small group of dedicated head and neck pathologists to reduce inter-observer variability, enable development of focused expertise and enable surgeons and pathologists to develop closer working relationships that would allow for a better understanding of mutual challenges in interpretation of these complex surgical specimens [7]. Studies that do demonstrate a relationship between margin status and poor survival outcomes did so using a dedicated pathologist in the interpretation of the specimens [3, 33, 34].

## Conclusion

This study demonstrated that in a cohort of patients with OSCC, those with close surgical margins had similar rates of recurrence and survival to those with clear margins. One possible explanation for this finding is that outcomes are equal, whether margins are close or widely clear, keeping in mind that patients with close margins were more likely to receive adjuvant treatment. Variability in specimen handling and interpretation is an alternative explanation for this finding.

## Data Availability

The datasets used and/or analysed during the current study are available from the corresponding author on reasonable request.

## Abbreviations

OSCC: Oral Squamous Cell Carcinoma
RFS: Recurrence-free survival
DSS: Disease-specific survival
ECS: Extracapsular Spread
PNI: Perineural Invasion
LVI: Lymphovascular Invasion
PH: Proportional Hazard
RT: Radiotherapy

## References

[1] Binahmed A, Nason RW, Abdoh AA (2007). The clinical significance of the positive surgical margin in oral cancer. Oral Oncol.

[2] Chen T-C, Wang C-P, Ko J-Y, Yang T-L, Lou P-J (2012). The impact of pathologic close margin on the survival of patients with early stage oral squamous cell carcinoma. Oral Oncol.

[3] Kurita H, Nakanishi Y, Nishizawa R, Xiao T, Kamata T, Koike T (2010). Impact of different surgical margin conditions on local recurrence of oral squamous cell carcinoma. Oral Oncol Pergamon.

[4] Nason RW, Binahmed A, Pathak KA, Abdoh AA, Sándor GKB (2009). What is the adequate margin of surgical resection in Oral cancer? Oral surgery, Oral medicine, Oral pathology, Oral radiology, and Endodontology. Mosby.

[5] Thomas J, Ow JNM (2011). Current Management of Advanced Resectable Oral Cavity Squamous Cell Carcinoma. Clin Exp Otorhinolaryngol.

[6] Kujan O, Khattab A, Oliver RJ, Roberts SA, Thakker N, Sloan P (2007). Why oral histopathology suffers inter-observer variability on grading oral epithelial dysplasia: an attempt to understand the sources of variation. Oral Oncol Pergamon.

[7] pathology JBAIA (1999). Surgical excision margins: a pathologist’s perspective.

[8] Fischer DJ, Epstein JB, Morton TH, Schwartz SM (2004). Interobserver reliability in the histopathologic diagnosis of oral pre-malignant and malignant lesions. J Oral Pathol Med.

[9] Edge SB, Compton CC (2010). The American joint committee on cancer: the 7th edition of the AJCC cancer staging manual and the future of TNM. Ann Surg Oncol.

[10] Jones KR, Lodge-Rigal RD, Reddick RL, Tudor GE, Shockley WW (1992). Prognostic factors in the recurrence of stage I and II squamous cell cancer of the Oral cavity. Arch Otolaryngol Head Neck Surg.

[11] Barry C, Shaw R, Woolgar J, Rogers S, Lowe D, Brown J (2013). OP081: evidence to support: a 3 mm margin as oncologically safe in early oral SCC. Oral Oncol Pergamon.

[12] Chen T-C, Chang H-L, Yang T-L, Lou P-J, Chang Y-L, Ko J-Y (2019). Impact of dysplastic surgical margins for patients with oral squamous cell carcinoma. Oral Oncol Pergamon.

[13] Mitchell DA, Kanatas A, Murphy C, Chengot P, Smith AB, Ong TK (2018). Margins and survival in oral cancer. Br J Oral Maxillofac Surg.

[14] Yamada S, Kurita H, Shimane T, Kamata T, Uehara S, Tanaka H (2016). Estimation of the width of free margin with a significant impact on local recurrence in surgical resection of oral squamous cell carcinoma. Int J Oral Maxillofac Surg.

[15] Wong LS, McMahon J, Devine J, McLellan D, Thompson E, Farrow A (2012). Influence of close resection margins on local recurrence and disease-specific survival in oral and oropharyngeal carcinoma. Br J Oral Maxillofac Surg.

[16] Ch’ng S, Burns SC, Stanton N, Gao K, Shannon K, Clifford A (2013). Close margin alone does not warrant postoperative adjuvant radiotherapy in oral squamous cell carcinoma. Cancer.

[17] Brandwein-Gensler M, Teixeira MS, Lewis CM, Lee B, Rolnitzky L, Hille JJ (2005). Oral squamous cell carcinoma: histologic risk assessment, but not margin status, is strongly predictive of local disease-free and overall survival. Am J Surg Pathol.

[18] van Es RJJ, van Nieuw AN, Slootweg PJ, Egyedi P (1996). Resection margin as a predictor of recurrence at the primary site for T1 and T2 Oral cancers: evaluation of Histopathologic variables. Arch Otolaryngol Head Neck Surg.

[19] Liao C-T, Chang JT-C, Wang H-M, Ng S-H, Hsueh C, Lee L-Y (2008). Analysis of risk factors of predictive local tumor control in Oral cavity cancer. Ann Surg Oncol Springer-Verlag.

[20] Tasche KK, Buchakjian MR, Pagedar NA, Sperry SM (2017). Definition of “close margin” in Oral cancer surgery and Association of Margin Distance with Local Recurrence Rate. JAMA Otolaryngol Head Neck Surg.

[21] Zanoni DK, Migliacci JC, Xu B, Katabi N, Montero PH, Ganly I (2017). A proposal to redefine close surgical margins in squamous cell carcinoma of the Oral tongue. JAMA Otolaryngol Head Neck Surg.

[22] Gokavarapu S, Chander R, Parvataneni N, Puthamakula S (2014). Close margins in Oral cancers: implication of close margin status in recurrence and survival of pT1N0 and pT2N0 Oral cancers. Int J Surg Oncol Hindawi.

[23] Johnson RE, Sigman JD, Funk GF, Robinson RA, Hoffman HT (1997). Quantification of surgical margin shrinkage in the oral cavity. Head Neck.

[24] Pindborg JJ, Reibel J, Holmstrup P (1985). Subjectivity in evaluating oral epithelial dysplasia, carcinoma in situ and initial carcinoma. J Oral Pathol Med.

[25] Meier JD, Oliver DA, Varvares MA (2005). Surgical margin determination in head and neck oncology: current clinical practice. The results of an international American head and neck society member survey. Head Neck.

[26] Kujan O, Oliver RJ, Khattab A, Roberts SA, Thakker N, Sloan P (2006). Evaluation of a new binary system of grading oral epithelial dysplasia for prediction of malignant transformation. Oral Oncol Pergamon.

[27] Abbey LM, Kaugars GE, Gunsolley JC, Burns JC, Page DG, Svirsky JA (1995). Intraexaminer and interexaminer reliability in the diagnosis of Oral epithelial dysplasia. Oral Surg Oral Med Oral Pathol Oral Radiol Endod.

[28] De Vet HCW, Knipschild PG, Schouten HJA, Koudstaal J, Kwee W-S, Willebrand D (1992). Sources of interobserver variation in histopathological grading of cervical dysplasia. J Clin Epidemiol Pergamon.

[29] Karabulut A, Reibel J, Therkildsen MH, Praetorius F, Nielsen HW, Dabelsteen E (1995). Observer variability in the histologic assessment of oral premalignant lesions. J Oral Pathol Med.

[30] Izumo T (2011). Oral premalignant lesions: from the pathological viewpoint. Int J Clin Oncol.

[31] Tilakaratne WM, Sherriff M, Morgan PR, Odell EW (2011). Grading oral epithelial dysplasia: analysis of individual features. J Oral Pathol Med.

[32] Nankivell P, Williams H, Matthews P, Suortamo S, Snead D, McConkey C (2013). The binary Oral dysplasia grading system: validity testing and suggested improvement. Oral Surg Oral Med Oral Pathol Oral Radiol.

[33] Sutton DN, Brown JS, Rogers SN, Vaughan ED, Woolgar JA (2003). The prognostic implications of the surgical margin in Oral squamous cell carcinoma. Int J Oral Maxillofac Surg.

[34] Dik EA, Willems SM, Ipenburg NA, Adriaansens SO, Rosenberg AJWP, van Es RJJ (2014). Resection of early oral squamous cell carcinoma with positive or close margins: relevance of adjuvant treatment in relation to local recurrence: margins of 3 mm as safe as 5 mm. Oral Oncol Pergamon.

